# Expression of tumor antigens on primary ovarian cancer cells compared to established ovarian cancer cell lines

**DOI:** 10.18632/oncotarget.10028

**Published:** 2016-06-14

**Authors:** Kamila Kloudová, Hana Hromádková, Simona Partlová, Tomáš Brtnický, Lukáš Rob, Jiřina Bartůňková, Michal Hensler, Michael J. Halaška, Radek Špíšek, Anna Fialová

**Affiliations:** ^1^ Department of Immunology, Charles University, 2nd Faculty of Medicine, University Hospital Motol, Prague, Czech Republic; ^2^ Department of Obstetrics and Gynaecology, Charles University, 2nd Faculty of Medicine, University Hospital Motol, Prague, Czech Republic; ^3^ Research Department, Sotio, Prague, Czech Republic

**Keywords:** high-grade serous epithelial ovarian cancer, tumor-associated antigens, immunotherapy, cancer cell lines

## Abstract

In order to select a suitable combination of cancer cell lines as an appropriate source of antigens for dendritic cell-based immunotherapy of ovarian cancer, we analyzed the expression level of 21 tumor associated antigens (BIRC5, CA125, CEA, DDX43, EPCAM, FOLR1, Her-2/neu, MAGE-A1, MAGE-A2, MAGE-A3, MAGE-A4, MAGE-A6, MAGE-A10, MAGE-A12, MUC-1, NY-ESO-1, PRAME, p53, TPBG, TRT, WT1) in 4 established ovarian cancer cell lines and in primary tumor cells isolated from the high-grade serous epithelial ovarian cancer tissue. More than 90% of tumor samples expressed very high levels of CA125, FOLR1, EPCAM and MUC-1 and elevated levels of Her-2/neu, similarly to OVCAR-3 cell line. The combination of OV-90 and OVCAR-3 cell lines showed the highest overlap with patients' samples in the TAA expression profile.

## INTRODUCTION

Ovarian cancer is one of the most frequent malignancies with the highest mortality rate among all gynecological tumors [1, 2]. Rapid expansion of the disease and the lack of highly sensitive and specific biomarkers allowing for early diagnosis account for the fact that about 70% of patients are diagnosed at the stage of advanced and disseminated disease with poor prognosis [3].

Surgery and chemotherapy based on platinum and taxane derivates represent the standard treatment modalities for ovarian cancer [4]. Although over 80% of patients are highly responsive to the frontline treatment, the persistence of a small number of resistant tumor cells (minimal residual disease), leads to relapse in 60-70% of patients within 2-5 years [5, 6]. Induction of anti-tumor immune response might represent an additional treatment modality leading to the stabilization or slowing down of the tumor growth at the stage of minimal residual disease.

In order to design appropriate cancer immunotherapy strategies, it is important to comprehensively characterize antigenic profile of primary ovarian cancer cells and to understand the relevance of individual tumor antigens for the induction of efficient immune response. In this study, we analyzed the expression level of 21 tumor associated antigens on ovarian cancer cells isolated from the tumor tissue resected during the radical surgery for the serous epithelial ovarian cancer, which is the most common histological subtype of the diagnosed cases. We compared the results obtained on primary tumor cells with 4 ovarian cancer cell lines in order to select the most suitable cell line combination for use in dendritic cell (DC)-based cancer immunotherapy protocols described in Podrazil et al., 2015 [7]. We also evaluated the presence of tumor antigen specific antibodies in the peripheral blood of tested patients and the relationship between the TAA expression and progression-free survival.

## RESULTS

### Expression of tumor associated antigens on primary ovarian cancer cells, selected cell lines and PBMC

**Figure 1:**
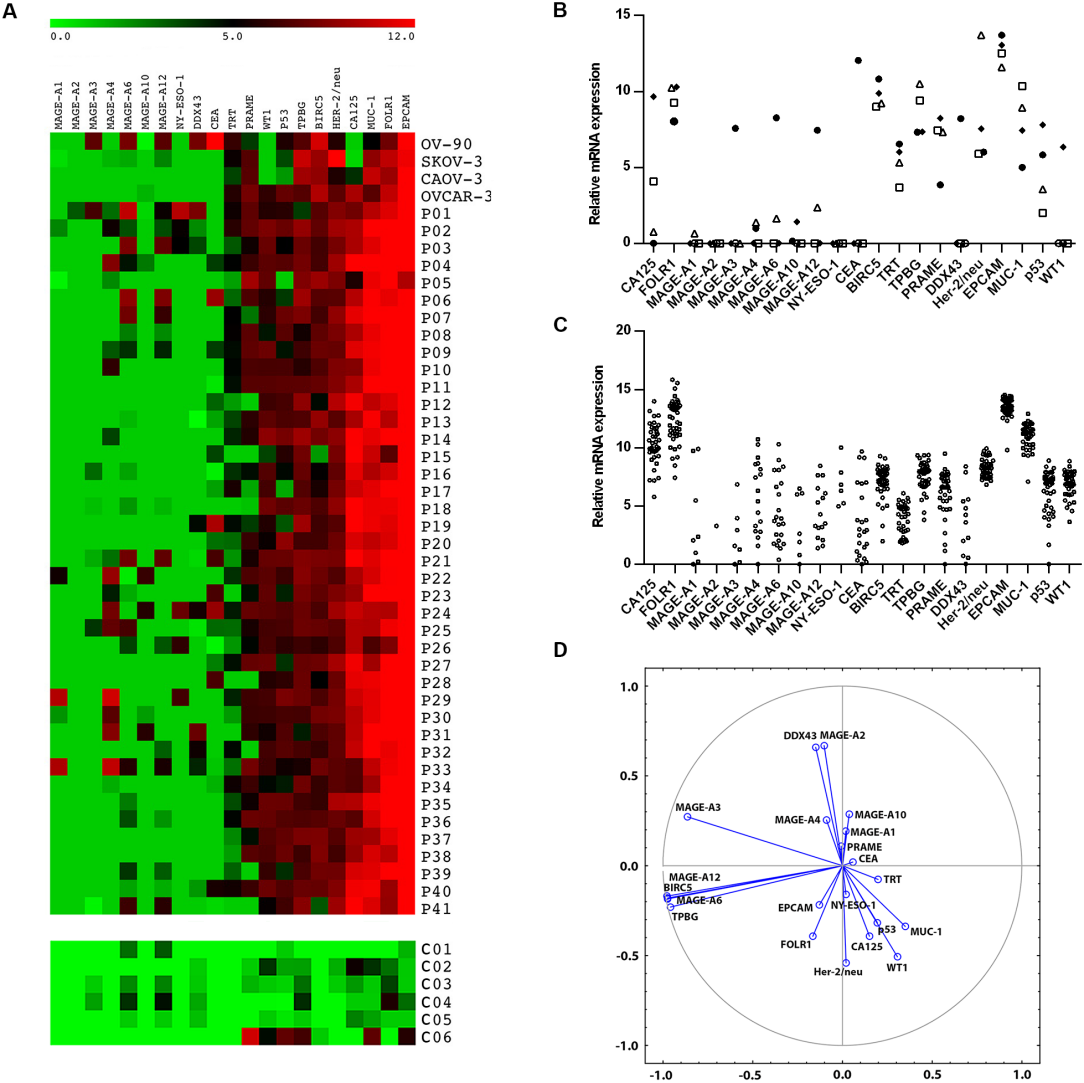
Relative mRNA expression of twenty one TAAs (BIRC-5, CA-125, CEA, DDX-43, EpCAM, FBP, HER-2/neu, MAGE-A1, MAGE-A2, MAGE-A3, MAGE-A4, MAGE-A6, MAGE-A10, MAGE-A12 NY-ESO-1, PRAME, p53, TPBG, TRT, WT-1) in cancer cell lines, primary tumor cells and control ovarian tissue (C01 – C06) Results were normalized to the expression of reference β-actin. Data are expressed as a heat map **A.**, relative mRNA expression of TAAs in cell lines (**B.** • OV-90, □ CAOV-3, Δ SKOV-3, ♦ OVCAR-3) and patients **C. D.** represents visualization of the TAA clusters extracted using Principal Component Analysis.

We measured the expression of mRNA levels of 21 TAAs, namely BIRC5, CA125, CEA, DDX43, EPCAM, FOLR1, Her-2/neu, MAGE-A1, MAGE-A2, MAGE-A3, MAGE-A4, MAGE-A6, MAGE-A10, MAGE-A12, MUC-1, NY-ESO-1, PRAME, p53, TPBG, TRT and WT1, on isolated serous epithelial ovarian cancer cells and compared the obtained data to the expression profiles of 4 estabilished ovarian cancer cell lines, OV-90, SKOV-3 OVCAR-3 and CAOV-3. Additionally, we evaluated the expression levels of TAAs on PBMCs obtained from patients and healthy controls. As expected, there was significant variability in the pattern and expression levels of investigated antigens between tested ovarian cancer cell lines and tumor cells obtained from patients (Figure 1).

Most of the tumor samples analyzed (> 90%) expressed very high levels of CA125, FOLR1, EPCAM and MUC-1 and intermediate levels of Her-2/neu, similarly to the OVCAR-3 cell line. All of these antigens showed significantly higher expression levels in tumor tissues in comparison to control ovarian tissue samples (p < 0.01). WT1 and p53, which are considered as biomarkers for high-grade serous ovarian carcinoma, were expressed in 82.9% and 70.7% of samples, respectively. Additionally, intermediate levels of PRAME, TPBG and BIRC5 were detected in > 60% of patients' samples. The expression level of BIRC5 was significantly higher in tumor tissues than in control ovarian samples (p = 0.015). From the cell lines analyzed, OV-90 and OVCAR-3 together cover the highest proportion of TAAs expressed on patients' samples (76.2%). The expression profile of TAAs on PBMCs did not differ between the patient group and the controls (Figure S1).

### Pattern of tumor associated antigens expression

**Figure 2:**
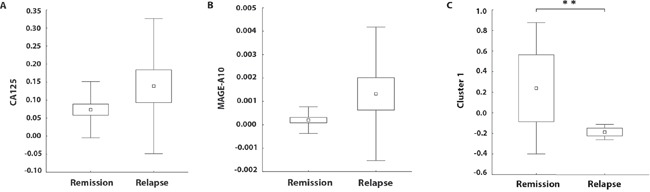
Relative mRNA expressions of CA125, MAGE-A10 and a Cluster 1 (MAGE-A3, MAGE-A6, MAGE-A12, BIRC-5 and TPBG) with regard to the progression-free survival The boundaries of the box indicate the S.E.M., and the lines in the box represent the mean. Whiskers indicate the SD. ** p < 0.01 (Mann-Whitney U test).

Statistical analysis showed that MAGE-A3, MAGE-A6, MAGE-A12, BIRC5 and TPBG were co-expressed in a cluster. The correlations among the expression levels of MAGE-A6, MAGE-A12, BIRC5 and TPBG are very strong, with r > 0.99 and p < 0.001. Correlations between MAGE-A3 and the other 4 TAAs are also highly significant, with r > 0.70 and p < 0.001. Other cluster was formed by MAGE-A2 with DDX43 (r = 0.81; p < 0.001) (Figure 1D). Patients who subsequently relapsed showed markedly higher levels of MAGE-A10 and CA125 expression (p = 0.09 and p = 0.08, respectively) in the primary tumor samples in comparison to patients who remained in remission (Figure 2). Indeed, 100% of patients with high expression levels of MAGE-A10 (n = 3), as shown in Figure 1A, underwent a relapse. Additionally, we have observed a significant decrease (p < 0.01) in the expression level of the Cluster 1 (MAGE-A3, MAGE-A6, MAGE-A12, BIRC-5 and TPBG) in relapsed patients in comparison with the patients in remission (Figure 2C).

### Detection of tumor antigen specific antibodies

Additionally, the presence of IgG antibodies against NY-ESO-1, Her-2/neu, MAGE-A3, MAGE-A4, MAGE-A10 and CA125 was analyzed in patients'sera. We only detected antibodies against NY-ESO-1, namely in 7.5% of patients. The titer of NY-ESO-1 antibodies (expressed as OD) significantly positively correlated with the level of expression of NY-ESO-1 mRNA.

## DISCUSSION

The profile of TAAs expressed on primary tumor samples is known to be very heterogenous. To select the suitable cell lines for DC-based ovarian cancer immunotherapy protocols, we analyzed the TAA profile of 41 fresh high-grade serous ovarian tumor samples and 4 established ovarian cancer cell lines. The combination of OV-90 and OVCAR-3 cell lines covered the highest proportion of antigens most frequently expressed on primary tumor samples.

As expected, most of the tumor samples analyzed (> 90%) expressed very high levels of CA125, FOLR1, EPCAM and MUC-1 and elevated levels of Her-2/neu, similarly to OVCAR-3 cell line. All of these antigens showed significantly higher expression levels in tumor tissues in comparison to control ovarian tissue samples. The most frequently tested ovarian cancer-associated antigen, the surface glycoprotein CA125, is usually evaluated in serum samples as a marker of disease progression, although with a limited specificity [8, 9]. In our study, markedly higher levels of CA125 and MAGE-A10 were detected in patients who underwent a relapse in comparison to the patients in remission. However, due to the prospective design of this study, we were not able to confirm these TAAs as independent prognostic factors via Cox proportional hazard model so far. Additionally, as the proportion of samples overexpressing MAGE-A10 was very low (n = 3), it is essential to analyze a larger cohort of patients to validate MAGE-A10 as a potential negative prognostic marker.

All of these antigens are promising targets for immunotherapy. EPCAM, which is known to be a stable antigen expressed in primary, metastatic as well as recurrent disease [10], is an intensively studied target of passive immunotherapy mediated by monoclonal antibodies, such as catumaxomab and catumaxomab/Removab^®^ [11]. Similarly, anti-CA125 monoclonal antibodies (Abagovomab, Oregovomab) and anti-FRα monoclonal antibody (Farletuzumab) are tested in clinical studies with promising results [11]. Additionally, FRα -specific cytotoxic T lymphocytes (CTL) were detected in malignant ascites [12, 13] as well as peripheral blood [14] of ovarian cancer patients, thus indicating that FRα peptides are immunogenic *in vivo*. Consequently, FRα -loaded DC vaccines were reported to be on trial recently [11]. Similarly, MUC-1 specific tumor infiltrating/associated CTL were detected in ovarian cancer patients [15]. Therapies targeting MUC-1 consists of monoclonal antibodies, peptide, carbohydrate, DNA, and dendritic cell vaccines, or small molecules (aptamers).

From the group of antigens frequently expressed at intermediate levels, p53, WT1 and Her-2/neu have been currently tested in clinical trials as promising targets of ovarian cancer immunotherapy. Rahma et al (2011) [16] tested in phase II trial two formulations of wt p53:264-272 peptide – subcutaneous administration of the peptide admixed with Montanide and GM-CSF or intravenous administration of peptide-pulsed DCs. Results showed that p53-specific immune responses could be generated with either protocol. Amplification of the Her-2/neu gene and over-expression of the Her-2/neu protein have been reported in a number of different tumor types including breast, colon, gastric, ovarian, esophageal and endometrial. For some of these cancers, anti-Her-2/neu treatment has become an essential part of the therapy A Phase I study showed that vaccination with Her-2/neu peptides could induce a strong and long lived CD4 and CD8 specific response [17]. Similarly to Her-2/neu, WT1 is expressed in a wide spectrum of hematological and solid tumors, including high-grade serous ovarian cancer. Kobayashi et al. (2014) [18] reported that WT1-pulsed DC – based vaccine was able to generate specific T cell responses in patients with recurrent ovarian cancer; however, there was no correlation between the observed immune response and overall survival time. Two from the antigens expressed at intermediate levels, namely BIRC5 and TPBG, were co-expressed in a cluster together with MAGE-A3, MAGE-A6 and MAGE-A12 (Cluster 1). Surprisingly, patients in remission expressed significantly higher levels of Cluster 1 than patients who underwent a relapse. However, the prognostic significance of Cluster 1 remains to be confirmed using Cox proportional hazard model after the follow-up care endpoint.

We have documented a heterogeneous and individual pattern of tumor antigen expression on primary tumor cells. This underlines the importance of the choice of the appropriate spectrum of tumor antigens in cancer immunotherapy trials. Ideal tumor antigen should be unanimously expressed on all tumor cells. However, none of the antigens analyzed in this study fulfills this criterion. This argues for the use of multiple tumor antigen approaches for the cancer immunotherapy of the ovarian cancer. Such strategy, if successful, would lead to the development of a complex immune response targeting different proteins and enhancing the chance of a long term control of tumor progression. Ovarian cancer cell lines selected in our study as the most suitable for DC-based immunotherapy protocols of high-grade ovarian cancer, namely OV-90 and OVCAR-3, cover together 76.2% of measured TAAs, including the most promising immunotherapy targets. Phase I/II clinical trial in metastatic prostate cancer patients treated with docetaxel combined with vaccines of LNCaP prostate cancer cell line – pulsed DCs showed induction and maintenance of PSA specific T cells and longer than expected survival [7]. Phase II clinical trial regarding the efficacy of the vaccine based on the same protocol to treat ovarian cancer is already in progress (NCT02107937).

## MATERIALS AND METHODS

### Patients

Peripheral blood and primary tumor tissue were obtained from 41 patients in stage III – IV of high-grade serous epithelial ovarian cancer, undergoing initial cytoreductive surgery at the University Hospital Motol in Prague. None of the patients enrolled in the study had received neoadjuvant chemotherapy prior to the surgery. Peripheral blood mononuclear cells (PBMC) and sera obtained from healthy donors were used as controls. Control ovarian tissue was obtained from patients undergoing surgery for ovarian cysts. Control C06 was human adult ovary full-length cDNA template obtained from Biosettia (San Diego, CA). All tissue specimens were collected with patient consent, and the study was approved by the Institutional Review Board of the University Hospital Motol. The clinico-pathological characteristics of the patients are summarized in Table 1.

**Table 1:** Clinicopathological characteristics of the EOC patients in the study

Variable	No.	%
Total no. of patients	41	
Age		
Mean	60	
Range	42-82	
FIGO stage		
III	41	100
Histological subtype		
High Grade Serous	41	100
Relapse		
Yes	17	41.5
No	22	53.7
ND	2	4.9

### Isolation of primary tumor cells and PBMC

Fresh tumor tissue was minced with scissors and digested in PBS containing 1 mg/ml of Collagenase D (Roche, Basel, Switzerland) at 37°C for 30 min, mechanically dissociated using the gentleMACS™ Dissociator (Miltenyi Biotec, Auburn, CA) and passed through a 100 μm nylon cell strainer (BD Biosciences, Franklin Lakes, NJ). Tumor cells were enriched using Ficoll-Paque density gradient solution (GE Healthcare, Waukesha, WI). Equally, PBMC from blood samples were isolated using Ficoll-Paque density gradient solution (GE Healthcare).

### Cell lines

The ovarian cancer cell lines OV-90, SKOV-3 and OVCAR-3 obtained from ATCC were cultured in RPMI 1640, CAOV-3 was cultured in DMEM. Culture media were supplemented with 10% heat inactivated FBS + L-glutamine + penicillin-streptomycin (all from Lonza) at 37°C and 5% CO2.

### Flow cytometry analysis

Tumor cell suspensions were stained with specific antibodies against EPCAM and CD45 (BioLegend, San Diego, CA). Proportions of EPCAM+CD45- tumor cells in the final cell suspension were assessed by flow cytometry using BD FACS Aria and FlowJo software.

### RNA extraction

Cellular suspensions containing at least 80% of EPCAM positive cells were used for RNA extraction. In the case of lower purity, the tumor cells were enriched by EasySep® Human EpCAM Positive Selection Kit (STEMCELL Technologies, Vancouver, Canada).

Total RNA was prepared from enriched tumor cell suspension using RNeasy mini or micro kit (Qiagen, Hilden, Germany). The DNase I (Qiagen, Hilden, Germany) treatment was performed during isolation. RNA concentrations were determined with a NanodropVC 2000c UV-Vis spectrophotometer (Thermo Scientific).

### Reverse transcription and qPCR

RNA was transcribed into cDNA by iScript™ Reverse Transcription Supermix (BIO-RAD) according to manufacturer's instructions.

The identity of qPCR products in each assay was verified by sequencing. Levels of various transcripts were evaluated by quantitative real-time PCR (qPCR) using CFX96 Real-Time System (BIO-RAD) and Kapa Probe Fast qPCR kit in the presence of specific primers and TaqMan probes obtained from TIB- MOLBIOL (Berlin, Germany). PCR conditions were 95°C for 3 min and 50 cycles of 95°C for 15 s and 60°C for 1min. We measured the mRNA expression for twenty one TAAs (BIRC5, CA125, CEA, DDX43, EPCAM, FOLR1, Her-2/neu, MAGE-A1, MAGE-A2, MAGE-A3, MAGE-A4, MAGE-A6, MAGE-A10, MAGE-A12, MUC-1, NY-ESO-1, PRAME, p53, TPBG, TRT, WT1) and for β-actin used as a reference gene. The relative expression of the target genes were normalized to the level of β-actin. All values are presented as means ± SEM. Specificity of the amplified PCR product was assessed by sequencing. The sequences of primers pairs and probes are summarised in Table S1.

### Detection of tumor antigen specific antibodies

Recombinant proteins NY-ESO-1, Her-2/neu, MAGE-A4 (Origene, Rockville, US), CA125 (R&D Systems) and MAGE-A3, MAGE-A10 (Abnova, Taipei, Taiwan) were diluted in Carbonate Coating Buffer (Invitrogen, Prague, Czech Republic) to a final concentration of 1 μg/ml and coated to 96-well plates overnight at 4°C. Plates were blocked for 1 hour with Assay Buffer (Invitrogen, Carlsbad, CA). Patients and control sera diluted to 1:50, 1:100 and 1:200 were incubated in the antigen-coated wells for 2 h. Plates were then incubated with secondary antibody (goat polyclonal antibody to human IgG, Abcam, Cambridge, UK) for 1 hour. TMB (Invitrogen) was used as a substrate. Reaction was stopped after 20 minutes by adding Stop Solution (Invitrogen). Plates were immediately read with absorbance at 450 nm. As a positive control the Cytomegalovirus Glycoprotein B protein was used. The cutoff value designating positive reaction was assessed as the mean OD of 15 sera obtained from healthy controls (NHS) + 3SD.

### Statistical analysis

Statistical analyses were performed using Statistica® 10.0 software (StatSoft, Tulsa, OK). TAA clusters were extracted using Principal Components Analysis. Differences between tumor tissues and control samples, as well as differences between patients in remission and patients with a relapsed EOC were tested using Mann-Whitney U Test. The results were considered statistically significant when p < 0.05.

## SUPPLEMENTARY FIGURE AND TABLE




