# Supplementary material for: Expression of tumor antigens on primary ovarian cancer cells compared to established ovarian cancer cell lines
Source: Oncotarget. 2016 Jun 14;7(29):46120–6. doi: 10.18632/oncotarget.10028 (PMC5216785; doi:10.18632/oncotarget.10028)
Supplement: Supplementary file 1 [file oncotarget-07-46120-s001.pdf]

Expression of tumor antigens on primary ovarian cancer cells compared to established ovarian cancer cell lines

SUPPLEMENTARY FIGURE AND TABLE

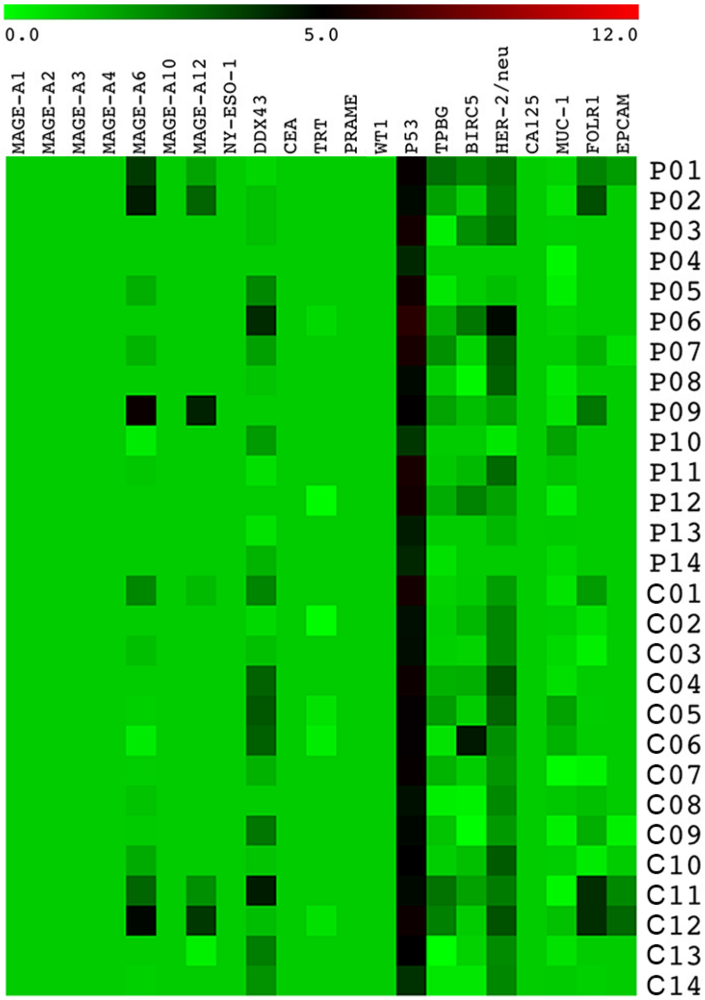

Supplementary Figure S1: Relative mRNA expression of twenty one TAAs (BIRC-5, CA-125, CEA, DDX-43, EpCAM, FBP, HER-2/neu, MAGE-A1, MAGE-A2, MAGE-A3, MAGE-A4, MAGE-A6, MAGE-A10, MAGE-A12 NY-ESO-1, PRAME, p53, TPBG, TRT, WT-1) on patients' (P) and healthy donors' (C) PBMCs. Results were normalized to the expression of reference β-actin. Data are expressed as a heat map.

Supplementary Table S1: The sequences of primers and probes used in this study

See Supplementary File 1
