# Supplementary material for: Expression of tumor antigens on primary ovarian cancer cells compared to established ovarian cancer cell lines
Source: Oncotarget. 2016 Jun 14;7(29):46120–6. doi: 10.18632/oncotarget.10028 (PMC5216785; doi:10.18632/oncotarget.10028)
Supplement: Supplementary file 2 [file oncotarget-07-46120-s002.docx]

**Table S1.** The sequences of primers and probes used in this study.

| **Name** | **FOR primer (5´-3´)** | **REV primer (5´-3´)** | **Probe (5´-3´)** |
| --- | --- | --- | --- |
| BIRC5 | GACCCGTTGGCAGAGGT | CCTCGGCCATCCGCTC | CCCGACGTTGCCCCCTGCCT |
| CA125 | CTCTGAGAGTGTGACCTCAAGAACAA | GTGGCAGGGGTCCAGTACC | ACCATCGGTCCTGGATCTCCACC |
| CEA | CAAATAATAACGGGACCTATGCCT | CAGGAGAAGTTCCAGATGCAGA | TTTGTCTCTAACTTGGCTACTGGCCGC |
| DDX43 | GATTTTGTTAGATGTGCGCCCA | GGTTCTTTCAAATAAGATTGTGCGAG | CGATGAACTGAATGAGGCCATGTAGCAC |
| EpCAM | GCAGGGTCTAAAAGCTGGTGT | ACCCATCTCCTTTATCTCAGCCTT | TGCTGTTATTGTGGTTGTGGTGATAGCAGT |
| FOLR1 | GCACCTCCTACACCTGCAAGA | CTCCCACTGCGCACTTGTTA | ACAAGGGCTGGAACTGGACTTCAGGGT |
| Her-2/neu | GGCGCTGGAGTCCATTCTC | TGGCCCCAAAAGTCATCAG | CCAGAGTGATGTGTGGAGTTATGGTGTGACTG |
| MAGE-A1 | TCTGAGGGACGGCGTAGAGT | TGAAAACCTTGCCTCCTCACA | CGGCCGAAGGAACCTGACCCA |
| MAGE-A2 | GTGGAGAGCCTGAGGTTCTGA | TGAAGACCCACAGGCAGATCT | TGACAAGTAGGACCCGAGGCACTGG |
| MAGE-A3 | CACTGAAGGAGAAGATCTGCCA | CTCTGCTCAAGAGGCATGATGA | CAGGGCAACAGGCGGGAGT |
| MAGE-A4 | CAACCGGAGGACAGGATTCC | GACCCACAGGCAGATCTTCTC | TGGAGGCCACAGAGGAGCACCA |
| MAGE-A6 | CGTCGGAAATTGGCAGTACTTC | CCAAAGACCAGCTGCAAGGA | TTCCTGTGATCTTCAGCAAAGCTTCCG |
| MAGE-A10 | GCTTGAGATCGGCTGAAGAGA | CACCCTCTGAGAGCAAGGTTCT | AGGCTCTGTGAGGAGGCAAGGGAGGT |
| MAGE-A12 | TCCGTGAGGAGGCAAGGTT | GCTCAAGTGGCATGATGACTCT | AGACAGGCCCCGGAGCAGCAC |
| MUC-1 | CGTAGCCCCTATGAGAAGGTTTC | GCGACGTGCCCCTACAAG | AGCAGCCTCTCTTACACAAACCCAGCA |
| NY-ESO-1 | AGCTGATGGAGAGCTGCAGTT | TCACTGTGTCCGGCAACATA | TGGTCTGCAGCAGTCAGTCGGATAGTC |
| PRAME | CGTTTGTGGGGTTCCATTC | CCAGAGGGAGGCAGGTG | TGGCTGTGTCTCCCGTCAAAGGC |
| p53 | GCTGCTCAGATAGCGATGGTCT | AAGGAAATTTGCGTGTGGAGTA | GGTACAGTCAGAGCCAACCTCAG |
| TPBG | GGTACTCCTGGGCTGGGT | GTGGGCACCTCGGTCAG | CGCTGCCGGACCAGTGCC |
| TRT | TTTCTACCGGAAGAGTGTCTGG | GCAGCTGCACCCTCTTCAA | TTGCAAAGCATTGGAATCAGACAGCAC |
| WT1 | TACACACGCACGGTGTCTTCA | CTCAGATGCCGACCGTACAAG | AGGCATTCAGGATGTGCGACGTGTG |
| β-actin | ATTGCCGACAGGATGCAGAA | GCTGATCCACATCTGCTGGAA | AGATCATTGCTCCTCCTGAGCGCA |
